# Inhibition of histamine receptor 3 suppresses glioblastoma tumor growth, invasion, and epithelial-to-mesenchymal transition

**DOI:** 10.18632/oncotarget.3672

**Published:** 2015-04-10

**Authors:** Jia-Ji Lin, Tian-Zhi Zhao, Wen-Ke Cai, Yong-Xiang Yang, Chao Sun, Zhuo Zhang, Yu-Qiao Xu, Ting Chang, Zhu-Yi Li

**Affiliations:** ^1^ Department of Neurology, Tangdu Hospital, The Fourth Military Medical University, Xi'an, China; ^2^ Department of Neurosurgery, Tangdu Hospital, The Fourth Military Medical University, Xi'an, China; ^3^ Department of Cardio-Thoracic Surgery, Kunming General Hospital of Chengdu Military Region, Kunming, China; ^4^ Department of Pathology, The Fourth Military Medical University, Xi'an, China

**Keywords:** histamine receptor 3, glioblastoma, epithelial-to-mesenchymal transition, invasion

## Abstract

Histamine receptor 3 (H3R) is expressed in various tumors and correlated with malignancy and tumor proliferation. However, the role of H3R in tumor invasion and epithelial to mesenchymal transition (EMT) remains unknown. Here, we explored the H3R in the highly invasive glioblastoma (GBM) and U87MG cells. We found that H3R mRNA and protein levels were up-regulated in the GBM and glioma cell lines compared to normal brain tissue and astrocytes. In U87MG cell line, inhibition of H3R by siRNA or the antagonist ciproxifan (CPX) suppressed proliferation, invasiveness, and the expression of EMT activators (Snail, Slug and Twist). In addition, expression of epithelial markers (E-cadherin and ZO-1) was up-regulated and expression of mesenchymal markers (vimentin and N-cadherin) was down-regulated *in vitro* and *in vivo* in a xenograft model. In addition, we also showed that inhibition of H3R by siRNA or CPX inactivated the PI3K/Akt and MEK/ERK signaling pathways, while inhibition of Akt or ERK activity with antagonists or siRNAs suppressed H3R agonist (*R*)-(α)-(−)- methylhistamine dihydrobromide (RAMH) mediated invasion and reorganization of cadherin-household. In conclusion, overexpression of H3R is associated with glioma progression. Inhibition of H3R leads to suppressed invasion and EMT of GBM by inactivating the PI3K/Akt and MEK/ERK pathways in gliomas.

## INTRODUCTION

Diffuse infiltration of tumor cells in the brain is a central hallmark of high-grade astrocytomas, especially glioblastoma (GBM) [1, 2]. Tumor invasiveness leads to severe structural and functional damage to the surrounding brain tissue, incomplete surgical resection and high frequency of tumor recurrence with a median survival time of 12–14 months [1, 2]. One important factor that contributes to the invasiveness of high-grade astrocytomas is the epithelial to mesenchymal transition (EMT) of the glioma cells [3]. EMT is a complex cellular process reflecting a high level of phenotypic plasticity, which is marked by the down-regulation of epithelial markers (E-cadherin and ZO-1) and transcriptional induction of mesenchymal markers (vimentin and N-cadherin) [4]. The transition of epithelial cells to mesenchymal cells induces the loss of cell-cell adhesion, cell polarity, and the acquisition of migratory and invasive properties [3]. Understanding the mechanisms that drive EMT is therefore important to identify new targets for the prevention of metastasis in astrocytoma.

Compared with the other three histamine receptors subtypes, histamine receptor 3 (H3R) is mostly expressed in neurons, in which regulates the synthesis and release of histamine and neurotransmitters as presynaptic autoreceptors and heteroreceptors [5–8]. Although it is widely considered to be specific for the central nervous system (CNS), H3R is also expressed in a variety of cancers, suggesting a possible role tumorigenesis. Expression of H3R in breast carcinomas is correlated with the levels of proliferating cell nuclear antigen (PCNA) level and an increased level of malignancy [9]. Expression of H3R in malignant adrenocortical cancer samples is also up-regulated compared to normal tissues [10]. Additional studies confirmed that the upregulated expression of H3R correlates with tumor progression. Activation of H3R by its agonist induces the proliferation and migration of pancreatic carcinoma PANC-1 cell and breast carcinomas MDA-MB-231 cells [9, 11]. However, it remains to be determined whether H3R is expressed in astrocytomas and whether H3R is involved in the regulation of invasiveness and the induction of EMT in these cells.

H3R functions as a presynaptic autoreceptor and heteroreceptor at numerous neurotransmission crossroads, and it regulates the release and synthesis of neurotransmitters, such as histamine, acetylcholine, dopamine, norepinephrine and 5-hydroxytryptamine [5–8]. It has been widely proven that H3R is involved in a series of neurological disorders, such as sleep disorders, Alzheimer's disease, schizophrenia and epilepsy [12, 13]. At the meanwhile, H3R has multiple isoforms and can target a variety of signal transduction pathways in the nervous system. For example, H3R activation not only inhibits the adenylyl cyclase (AC) pathway, but also results in phosphorylation of the mitogen-activated protein kinase (MAPK), phosphatidyl inositol 3-kinase (PI3K), calmodulin-dependent protein kinase II (CaMKII) and phospholipase A2 (PLA2) signaling pathways [13–16]. H3R blockade offers protection in several conditions such as inflammation, N-methyl-D-aspartic acid (NMDA)-induced neurotoxicity, ischemia-induced oxidative stress and autophagy [17–20]. Considering the high level of H3R expressionin the brain, its role in tumor progression, multiple signal transductions and general cerebral protection, we hypothesized that H3R may have a potential role in the regulation of the invasiveness and EMT of GBM.

In order to verify our hypothesis, the present studies were designed to (1) explore the H3R expression in the human astrocytoma tissues and cell lines by immunohistochemistry assay, real-time qPCR and western blot; (2) inhibit the overexpressed H3R by small-interfering RNA (siRNA) or antagonist in the U87MG cells to evaluate the role of H3R in the metastasis nature, EMT activators and markers in the U87MG cells; (3) explore the signaling pathways downstream of H3R by siRNAs or the selective agonist/antagonist in the U87MG cells; (4) explore the effects of knockdown or blockade of H3R on a xenografted model.

## RESULTS

### Expression of H3R correlates with the pathology grade of astrocytoma

To date, evidence for the expression of H3R in glioma was lacking and there was only one study reporting that H3R is not expressed in U373MG cells [21]. Here, 40 patients from Tangdu Hospital diagnosed with astrocytoma between 2008 and 2012 were selected, including 20 cases of low-grade astrocytoma (LGA: 10 cases of pilocytic astrocytoma (PA) and 10 cases of fibrillary astrocytoma (FA)) and 20 cases of high-grade astrocytoma (HGA: 10 cases of anaplastic astrocytoma (AA) and 10 cases of GBM). Ten samples of healthy normal brain tissue (NB) obtained from patients who underwent needle biopsy without malignancy served as control. The tissue samples were harvested and H3R expression was measured using immunohistochemistry, real-time PCR and western blot techniques.

**Figure 1:**
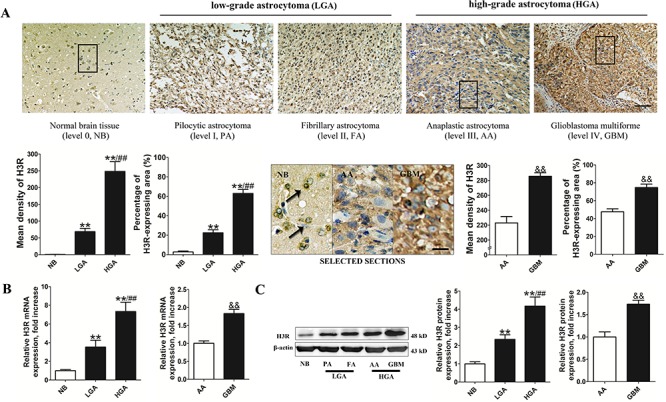
H3R was overexpressed in astrocytoma cells **A.** Representative pictures of immunohistochemical labeling for H3R expression in astrocytoma and NB (bar = 100 μm). The comparison image in SELECTED SECTIONS consists of the selected boxes from the upper panel (bar = 25 μm). **B and C.** Group results showing H3R expression from the RT-qPCR and western blot. Bars represent as mean ± SD. **P < 0.01 vs. NB; ^##^*P* < 0.01 vs. LGA; ^&&^*P* < 0.01 vs. AA; *n* = 10 for each grade of tissue samples.

Figure 1A shows that positive immunoreactivity of H3R was detected mostly in the soma of neurons in the NB tissues but not in the glia cells. In contrast, H3R expression was detected in glioma cells of astrocytoma tissues and was highest in the GBM tissue. Analysis using Image-Pro software showed that the percentage of expression area (PEA) and the mean density (MD) of H3R were significantly higher in the astrocytoma tissue than in the NB tissues, and both indexes were higher in the HGA than in the LGA (*P* < 0.01). In addition, the PEA and MD of H3R protein expression in the GBM tissue were significantly higher than in the AA tissues (*P* < 0.01). These results were confirmed using real-time PCR and western blot techniques (Figures 1B and 1C). We found that LGA and HGA tissues had higher mRNA and protein expression levels of H3R than NB tissues (*P* < 0.01). In addition, the relative level of H3R mRNA and protein expression in HGA was significantly higher than that in the LGA (*P* < 0.01). Both mRNA and protein expression of H3R in the GBM were also significantly higher than in the AA (*P* < 0.01).

### H3R regulates U87MG cell proliferation

**Figure 2:**
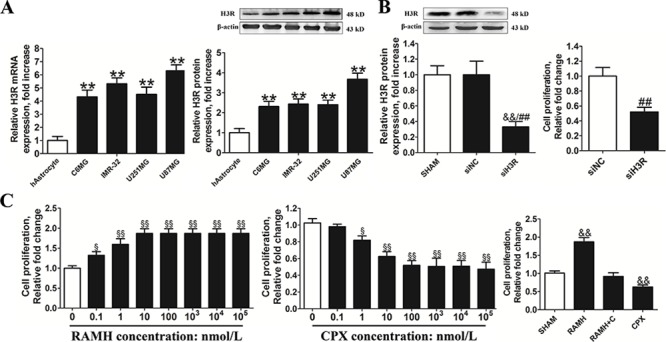
Inhibition of the H3R expression by siRNA or an antagonist decreased the proliferation of U87MG cells **A.** Group results of H3R expression from the RT-qPCR and western blot in the glioma cells and the human astrocytes. **B.** The effects of H3R siRNA on the proliferation of U87MG cells. The specific siRNA for H3R significantly decreased H3R protein expression in U87MG cells and cell proliferation in U87MG cells. **C.** The effects of H3R agonist/antagonist on the cell proliferation of U87MG cells. U87MG cells were incubated with H3R agonist/antagonist and cell proliferation was measured. The effects of 100 nmol/L RAMH on the proliferation of the U87MG cells were completely blocked by 100 nmol/L CPX. Bars represent as mean ± SD. **P < 0.01 vs. hAstrocyte; ^&&^*P* < 0.01 vs. SHAM; ^##^*P* < 0.01 vs. siNC; ^§^*P* < 0.05 vs. 0; ^§§^*P* < 0.01 vs. 0; *n* = 8 for each group.

H3R expression levels in the normal human astrocytes (hAstrocytes) and glioma cell lines (C6MG, IMR-32, U251MG, U87MG) were measured using real-time qPCR and western blot techniques. The results in Figure 2A showed that glioma cell lines had a significantly higher level of H3R mRNA expression than hAstrocytes. U87MG cells had the highest endogenous expression of H3R of the four glioma cell lines tested so it was selected for further *in vitro* experiments. In order to explore the role of H3R in these cells, a siRNA specific for H3R was employed. Western blot analysis of lysates of cells incubated with the H3R siRNAs (Figure 2B) showed that the knockdown efficiency of H3R was approximately 63% in U87MG cells (siH3R U87MG cells) compared to the negative control siRNA U87MG cells (siNC U87MG cells). Down-regulation of H3R expression was found to decrease cell proliferation in these cells as shown in Figure 2B (*P* < 0.01).

To further analyze the role of H3R, U87MG cells plated in 96-well plates (1.5 × 10^4^ cells/well) were incubated with the H3R selective agonist (*R*)-(α)-(−)-methylhistamine dihydrobromide (RAMH) or/and its antagonist ciproxifan (CPX). We found that pretreatment with the H3R selective agonist RAMH (10^−3^-10 μmol/L) promoted proliferation of U87MG cells in a dose-dependent manner. In contrast, blocking H3R activity with an antagonist CPX (10^−3^-10 μmol/L) suppressed proliferation of U87MG cells in a dose-dependent manner (Figure 2C). Cell proliferation of U87MG cells following incubation with 100 nmol/L RAMH was blocked by 100 nmol/L CPX. Based on this, 100 nmol/L RAMH and CPX were used in subsequent experiments.

### Inhibition of H3R expression suppresses metastasis in U87MG cells

**Figure 3:**
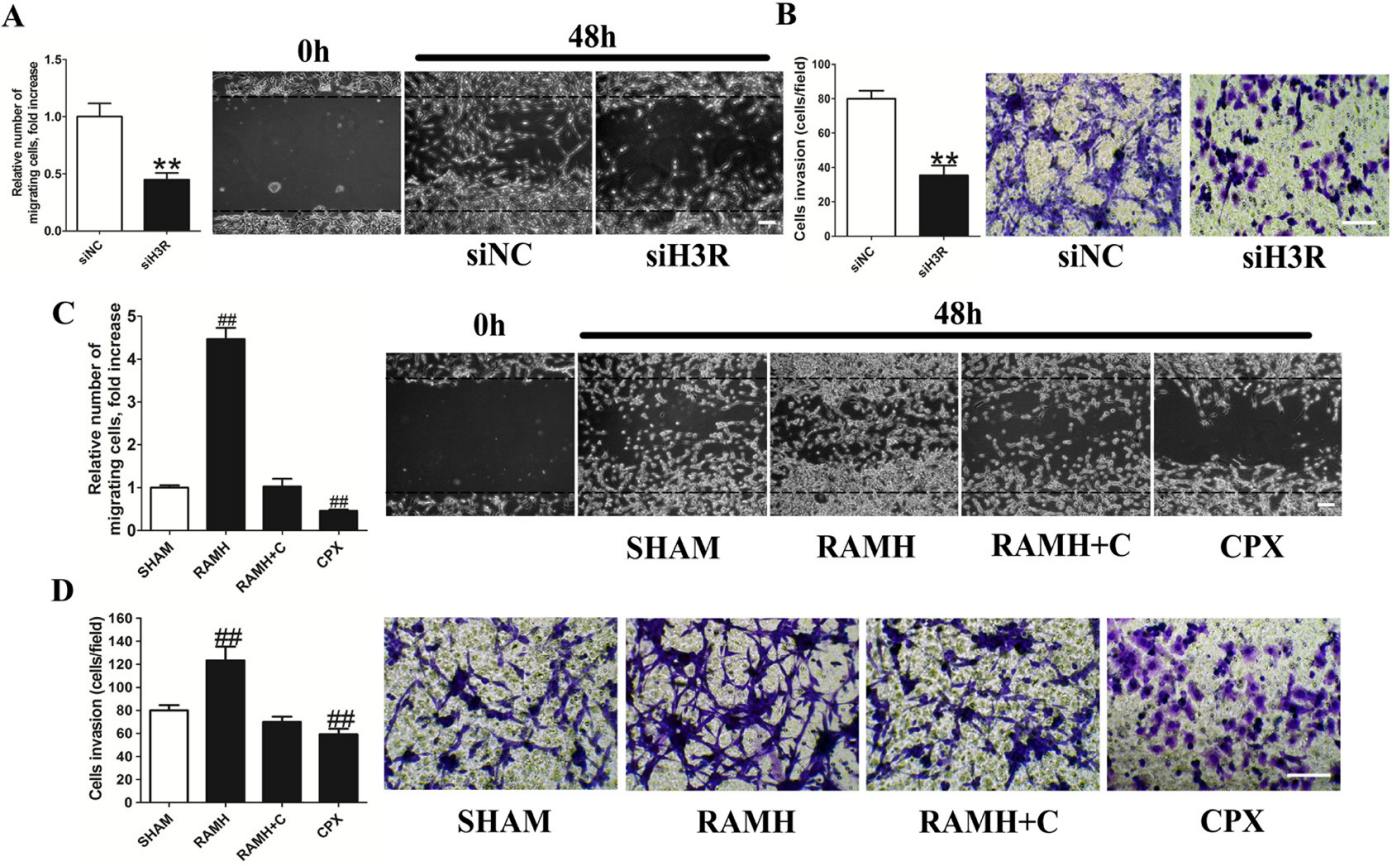
Inhibition of the H3R by the siRNA or CPX decreased the metastatic and invasive ability of U87MG cells **A and B.** Group results from the wound healing migration assay and transwell invasion assay after down-regulation of H3R by siRNA. H3R down-regulation by siRNA inhibited cell migration and invasion of U87MG cells. **C and D.** Group results from the wound healing migration assay and transwell invasion assay after pretreatment with H3R agonist RAMH or/and antagonist CPX. H3R blockade by antagonist inhibited cell migration and invasion of U87MG cells. Bars represent as mean ± SD. **P < 0.01 vs. siNC; ^##^*P* < 0.01 vs. SHAM; *n* = 8 for each group.

We used the wound healing migration and the transwell invasion assays to determine whether H3R can affect metastasis in U87MG cells (Figure 3A). We found that upon down-regulation of H3R expression using siRNA, migration of the U87MG cells towards artificially created wounds in confluent cell monolayers was suppressed as shown by the decreased number of migrating cells (*P* < 0.01). Down-regulation of H3R expression also resulted in fewer cells infiltrating the membranes in the transwell assay compared to the control (*P* < 0.01, Figure 3B).

A selective H3R agonist and antagonist were used in parallel in the wound healing and transwell assays to confirm our results as shown in Figure 3C. We found that H3R activation by RAMH induced a significant increase in migration compared to the control (*P* < 0.01), which was inhibited by CPX. In addition, blockade of H3R by CPX led to a sharp decline in migration (*P* < 0.01). As expected, RAMH increased the number of infiltrating U87MG cells in the transwell assay (*P* < 0.01, Figure 3D), an effect that was inhibited by co-incubation with CPX. Lastly, the antagonist CPX reduced the number of migrating cells (*P* < 0.01).

### Inhibition of H3R expression suppresses EMT in U87MG cells

**Figure 4:**
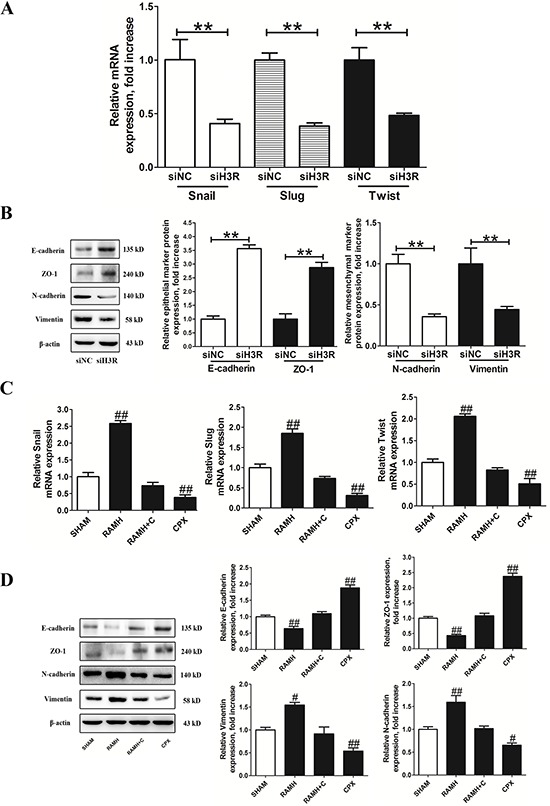
Inhibition of the H3R by the siRNA or CPX suppressed EMT progression in U87MG cells **A and B.** Group results from the RT-qPCR and western blots after down-regulation of H3R expression using the siRNA. H3R down-regulation by the siRNA decreased EMT activators mRNA, increased epithelial markers (E-cadherin and ZO-1) and decreased mesenchymal markers (N-cadherin and vimentin) in U87MG cells. **C and D.** Group results from the RT-qPCR and western blot after pretreatment with the H3R agonist RAMH or/and the antagonist CPX. Blockade of H3R by CPX decreased EMT activators mRNA, increased epithelial marker (E-cadherin and ZO-1) and decreased mesenchymal marker (N-cadherin and vimentin) in U87MG cells. Bars represent as mean ± SD. **P < 0.01 vs. siNC; ^#^*P* < 0.05 vs. SHAM; ^##^*P* < 0.01 vs. SHAM; *n* = 8 for each group.

The mRNA levels of the EMT-activators snail, slug and twist were measured in U87MG cells depleted of H3R. We found that all markers of EMT were significantly down-regulated compared with the control group (*P* < 0.01, Figure 4A). In parallel, H3R down-regulation also resulted in an up-regulation of the epithelial markers E-cadherin and ZO-1 and the down-regulation of the mesenchymal markers N-cadherin and vimentin (*P* < 0.01, Figure 4B).

Incubation with RAMH resulted in up-regulated mRNA expression of slug, snail and twist in U87MG cells (*P* < 0.01, Figure 4C), and this effect was blocked by CPX. Similarly, pretreatment with CPX resulted in down-regulated mRNA levels of EMT activators (*P* < 0.01, Figure 4C). Immunoblotting assays confirmed that the H3R selective agonist RAMH significantly increased the protein expression of N-cadherin and vimentin while decreasing E-cadherin and ZO-1 in U87MG cells (*P* < 0.01, Figure 4D). This effect was reversed by the selective H3R antagonist CPX. In addition, pretreatment with CPX up-regulated E-cadherin and ZO-1 expression and down-regulated N-cadherin and vimentin in the U87MG cells (*P* < 0.01, Figure 4D).

### H3R activates EMT via the PI3K/Akt and MEK/ERK signaling pathways

**Figure 5:**
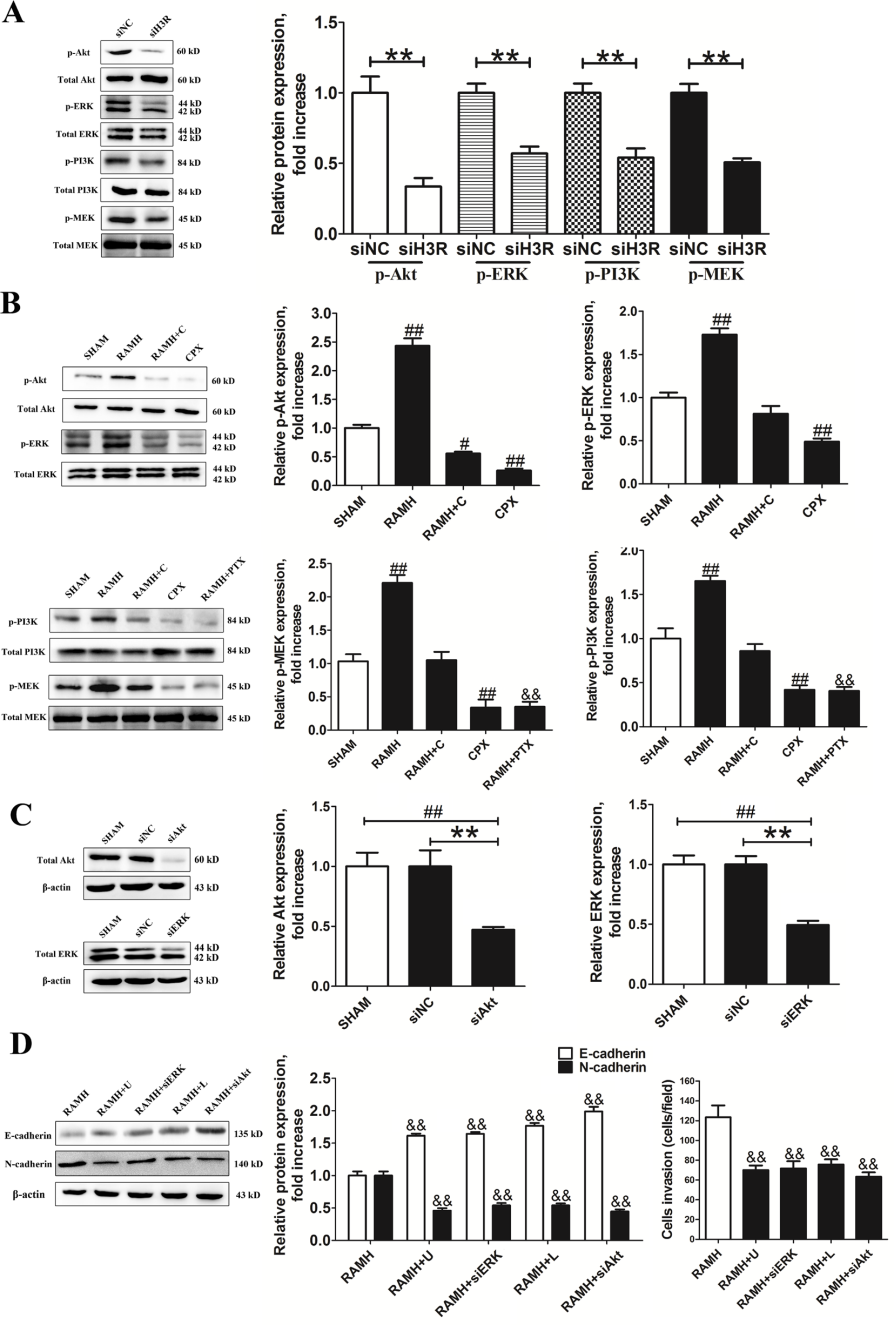
Inhibition of Akt/ERK by the siRNA or CPX suppressed RAMH-induced cell invasion or reorganization of the cadherin-household **A and B.** Representative immunoblots of samples from U87MG cell lysates subjected to different treatments and their quantitative densitometric analysis. Inhibition of H3R by the siRNA or CPX suppressed the activation of PI3K/Akt and MEK/ERK signaling pathways. **C and D.** Group results from western blot after application of Akt2/ERK1 siRNAs and antagonists. Applicantion of Akt2/ERK1 siRNAs significantly inhibited the Akt/ERK expression in the U87MG cells. Inhibition of Akt/ERK by siRNA or antagonist suppressed RAMH-induced cell invasion and reorganization of the cadherin-household; Bars represent as mean ± SD. **P < 0.01 vs. siNC; ^#^*P* < 0.01 vs. SHAM; ^##^*P* < 0.01 vs. SHAM; ^&&^*P* < 0.01 vs. RAMH; *n* = 8 for each group.

The Akt and ERK signaling pathways have been reported to play a role in modulating cell invasion and the progression of EMT. Here, we examined the effect of H3R on these two signaling pathways. We found that H3R down-regulation by siRNA significantly decreased the phosphorylation of PI3K, Akt, MEK and ERK, but not their total protein levels (*P* < 0.01, Figure 5A). Similarly, treatment of cells with the H3R-selective agonist significantly increased the phosphorylation level of Akt, PI3K, ERK and MEK, while the H3R antagonist CPX significantly suppressed phosphorylation of PI3K, Akt, MEK and ERK (*P* < 0.01). In both cases, the total protein levels of PI3K, Akt, MEK and ERK (*P* < 0.01, Figure 5B) remained unaffected as observed in untreated cells. Co-incubation with 100 nmol/L CPX suppressed 100 nmol/L RAMH induced phosphorylation of PI3K, Akt, MEK and ERK. In addition, 100 ng/mL pertussis toxin (PTX) pretreatment completely prevented the activation observed following RAMH treatment (*P* < 0.01). These observations indicated that the H3R mediated activation of PI3K/Akt and MEK/ERK signaling pathways in U87MG cells depended on PTX sensitive G_i/o_-proteins.

In order to verify the role of PI3K/Akt and MEK/ERK signaling pathways in the H3R related cell invasion and EMT progression, siRNAs for Akt2 or ERK1 were used and the knockdown efficiencies for Akt and ERK were found to be 53% and 51% respectively in U87MG cells (*P* < 0.01, Figure 5C). Down-regulation of Akt or ERK significantly attenuated the effects of RAMH on the protein levels of E-cadherin and vimentin compared to the negative control (*P* < 0.01, Figure 5D). The results of the transwell assay showed that knockdown of Akt or ERK suppressed the RAMH mediated increase in the number of infiltrating cells (*P* < 0.01, Figure 5D). The antagonists for PI3K/Akt and MEK/ERK signaling pathways, LY294002 and U-0126, were also found to suppress the RAMH induced increase in migration of U87MG cells (*P* < 0.01, Figure 5D). Correspondingly, LY294002 and U-0126 suppressed the RAMH induced up-regulation of N-cadherin and the down-regulation of E-cadherin (*P* < 0.01, Figure 5D).

### H3R inhibition inhibits tumor growth *in vivo*

**Figure 6:**
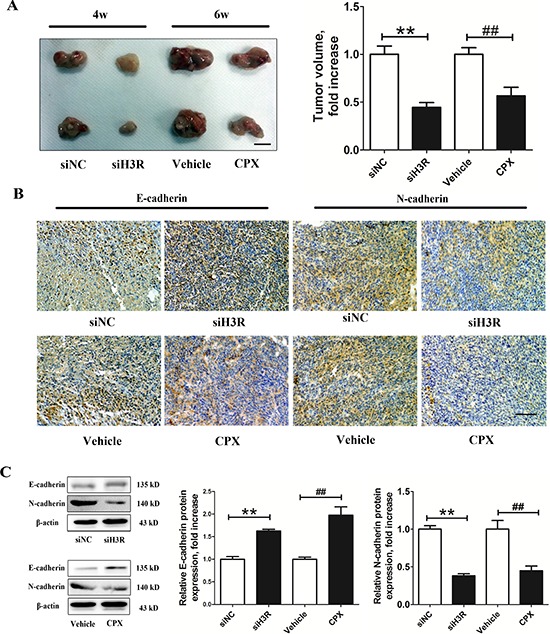
Inhibition of the H3R by the siRNA or CPX restrains the growth and EMT progression in a xenografted model **A.** Group representative images of xenograft tumors and quantitative tumor volume analysis after treatment; **B.** Group representative pictures of immunohistochemical labeling for the epithelial and mesenchymal phenotype markers in the harvested tumor tissues (bar = 100 μm). **C.** Western blot assay for the expression of the epithelial and mesenchymal phenotype markers in the harvested tumor tissues and their quantitative densitometric analysis. Bars represent as mean ± SD. **P < 0.01 vs. siNC; ^##^*P* < 0.01 vs. SHAM; *n* = 8 for each group.

To evaluate the effect of inhibition of H3R *in vivo*, we generated a subcutaneous xenograft model in nude mice. The rats were randomly assigned to one of the following four groups: (1) siNC group: nude rats were inoculated with the U87MG cells with mock-transfection for 4 w; (2) siH3R group: nude rats were inoculated with the U87MG cells transfected with H3R siRNAs for 4 w; (3) Vehicle group: nude rats were inoculated with the U87MG cells. When the subcutaneous tumors reached 50 mm^3^ (about 2 w), the nude rats received I.V. injections of vehicle (0.9% NaCl) once a day for 4 w; (4) CPX group: nude rats were inoculated with the U87MG cells. When the subcutaneous tumors reached 50 mm^3^ (about 2 w), the nude rats received I.V. injections of CPX (3 mg/kg) once a day for 4 w. After 4 w, the tumor volume in the siNC group is higher than that in the siH3R group (*P* < 0.01, Figure 6A). Successive abdominal administration of 3 mg/kg CPX for 4 w also induced a limited delay in tumor growth in the CPX group compared with the Vehicle group (*P* < 0.01, Figure 6A). Furthermore, immunohistochemistry studies of tumor sections revealed that E-cadherin expression was significantly increased and N-cadherin decreased in the siH3R group and CPX groups compared with the siNC group and Vehicle groups (Figure 6B). The western blot data of tumors in the siH3R and CPX groups also showed that the protein levels of E-cadherin increased significantly, while the mesenchymal phenotype marker N-cadherin decreased compared with the siNC and Vehicle groups (*P* < 0.01, Figure 6C).

## DISCUSSION

Expression of H3R has been reported for neurons [22–25]. More recent studies have reported expression of H3R in the cultured rat astrocytes from the cortex, cerebellum, hippocampus and striatum [26]. Consistent with these studies, our results showed that high levels of H3R expression are characteristic of neurons and lower levels are found in the glia cells of NB tissue and cultured hAstrocytes. In contrast, intense H3R staining was observed in the human glioma cells of astrocytoma tissue, especially in the GBM. Even though expression of H3R has not been documented for U373MG cells [21], examination of glioma cell lines (C6MG, IMR-32MG, U251MG, U87MG) revealed a relatively high level of both H3R mRNA and protein compared to hAstrocytes. This atypical up-regulation of H3R in the glioma cells may be a presentation of neuronal differentiation pattern in glioma tumors. Further analysis of H3R showed that HGA had elevated mRNA and protein expression, increased PEA and MD of H3R than the LGA. Therefore, H3R expression correlates with the pathology grade and H3R may be a prognosis factor in astrocytoma.

The up-regulated expression of H3R in the GBM compared to other grades of astrocytomas suggested a possible role in tumor progression of the GBM. In order to investigate the role of H3R in tumor progression, we used the H3R antagonist CPX and a specific siRNA for H3R *in vitro* and *in vivo* to down-regulate the activity or expression of the protein. Compared with other H3R antagonists, CPX exhibited a non-interactive antagonistic action with other histamine receptors and blockade of H3R by CPX showed protection in a series of pathologic conditions, such as schizophrenia, Alzheimer's disease and sleep disorders [27–29]. We found that inhibition of H3R by CPX or siRNA in U87MG cells significantly inhibited cell proliferation *in vitro* and *in vivo*, consistent with previous studies using the histamine receptor 1 and 2 in astrocytoma [21, 30]. Interestingly, a previous study showed that the effect of H3R on tumor proliferation was tumor-dependent. For example, activation of H3R by agonists induced the proliferation of pancreatic carcinoma PANC-1 cells and breast carcinoma MDA-MB-231 cells [9, 11], while activation of H3R by an agonist suppressed the proliferation of hepatoma McA-RH7777 cells and cholangiocarcinoma Mz-ChA-1 cells [31, 32]. The factors that dicided whether H3R was a stimulatory or inhibitory receptor on the tumor proliferation were complex, including the characteristics of tumors, H3R isoforms and the complexity of the signal transduction pathways involved. First, activation of H3R by exogenous histamine suppressed the proliferation of hepatoma McA-RH7777 cells [31], while H3R blockade promoted proliferation due to an unrespected release of cytochrome P450 enzymes within the McA-RH7777 cells, which regulated cell proliferation by controlling the levels of growth regulatory factors [12]. Second, it has been documented that H3R isoforms couple to diverse G-proteins to diversify their signaling effects in different cell systems [14, 33]. Specifically, H3R stimulation by RAMH (acting via Gα_i_) inhibited hyperplastic biliary growth by inhibition of cAMP [34], while RAMH (acting through Gα_o_) decreased the growth of cholangiocarcinoma Mz-ChA-1 cells by inositol triphosphate (IP_3_)/Ca^2+^, which is independent of cAMP activation [32]. Third, H3R activation not only inhibited the adenylyl cyclase-cAMP dependent pathway, but also activated the MAPK, PI3K, CaMKII, and PLA signaling pathways [13–16]. In different tumors, the major signaling pathways mediated by H3R may be different, and H3R may promote the opposite effect on one signaling in the different tumors. For example, H3R activation by RAMH promoted the MAPK signaling in the hyperplastic biliary [34], while H3R activaiton suppressed MAPK signaling in the cholangiocarcinoma Mz-ChA-1 cells [32]. Taken together, the final effect of H3R activation on the tumor proliferation needs repeated test for verification. Our findings disclosed no conflicts with the existing knowledge.

Even though the role of H3R in tumor proliferation has been extensively documented, it remained to be determined whether H3R is involved in the regulation of invasion. Here we showed for the first time that inhibition of H3R suppressed migration and invasion of glioma cells *in vitro*, while activation of H3R by an agonist had the opposite effect. Studies in GBMs showed that EMT (−like) processes are of clinical relevance in malignant brain tumors [35–37], which involved reorganization of the cadherin-household (switching from E-cadherin to N-cadherin) to break cell-to-cell contacts [38, 39]. Members of the Twist-, Slug- and Snail-family mediate increased GBM-cell motility and invasiveness both *in vitro* and *in vivo* as shown in animal studies and in patient-derived specimens [40–42]. In our studies, we found that knockdown of H3R expression suppressed the expression EMT-activators (Twist, Slug and Snail) and restored the expression of epithelial cell markers (E-cadherin and ZO-1) while blockade of its activity down-regulated the expression of mesenchymal cell markers (N-cadherin and Vimentin) in glioma U87MG cells. Taken together, inhibition of H3R expression or activity in glioma cells suppressed tumor invasion and reversed EMT, suggesting that targeting H3R signaling in GBM may be a promising anticancer strategy.

A possible explanation for the different tumor behaviors of GBM is the deregulation of signal transduction pathways such as the PI3K/Akt and MEK/ERK pathways caused by a large number of genetic abnormalities [43]. A large number of downstream molecules and crosstalk of these two signaling pathways exert a wide range of influences on GBM [43]. Previous studies reported that both the PI3K/Akt and MEK/ERK pathways are constitutively up-regulated in the majority of GBMs, which helps the unrestricted growth of glioma cells and enhances tumor invasion [44–47]. Moreover, the activation of these pathways plays a role in the progression of EMT [48, 49]. Here we report that upon downregulation of H3R expression or activity, activation of the PI3K/Akt and MEK/ERK signaling pathways was suppressed, while activation of H3R by RAMH induced activation of both signaling pathways in a PTX-sensitive G_i/o_ protein manner. In order to further demonstrate the role of above signaling pathways in the H3R related cell invasion and EMT progress, we applied the selective antagonist or we down-regulated the expression of signaling components of the PI3K/Akt and MEK/ERK pathways. Either knockdown or blockade of the PI3K/Akt or MEK/ERK signaling pathways inhibited RAMH-mediated increase in cell invasion and reorganization of the cadherin-household, confirming that PI3K/Akt and MEK/ERK signaling pathways play a role in the H3R-mediated tumor invasion and EMT progression.

Since histamine and histamine receptors were discovered, the evidence of their role in oncology, including astrocytoma. It has been suggested that there are multiple and context-dependent effects of H3R on proliferation in tumors. However, direct evidence of the expression of H3R in glioma or an understanding of its involvement in tumor invasion and EMT progress was lacking. Here we found that H3R was over-expressed in glioma cells and tissues and inhibition of H3R suppressed invasion and EMT in glioblastoma through the PI3K/Akt and MEK/ERK signaling pathways. The biological functions of H3R found in this study provided a basis for additional pathological and clinical investigations, suggesting that H3R may be a novel target for therapeutic intervention in GBM.

## MATERIALS AND METHODS

### Patient samples

The protocol for this study was approved by the ethics committee of the Fourth Military Medical University. Forty patients from the Tangdu Hospital diagnosed with astrocytoma between 2008 and 2012 were selected for the study. All patients involved in the study provided consent. Patients with other medical conditions or those who received treatment prior to surgery were also excluded. The pathological specimens were reviewed, and histological classifications of astrocytoma were made based on the World Health Organization (WHO) Classification of brain tumors [50]. We identified 20 cases of LGA (10 cases of PA and 10 cases of FA) and 20 cases of HGA (10 cases of AA and 10 cases of GBM). Ten healthy NB obtained from patients who underwent needle biopsy without malignancy served as controls. All the slides were re-examined by two pathologists to ensure correct diagnosis. Tissue samples were harvested for immunohistochemistry assay, real-time PCR and western blot analysis.

### Cell lines

Human glioma cell lines, C6MG, IMR-32, U87MG and U251MG cells were purchased from the American Type Culture Collection (Rockville) and the Cell Bank of the Chinese Academy of Sciences. Cells were cultured using Dulbecco's modified Eagle's medium (DMEM) supplemented with 10% heat-inactivated fetal bovine serum (FBS) and 1% penicillin-streptomycin antibiotics. Normal human astrocytes (hAstrocytes) were obtained from ScienCell and propagated in astrocyte medium (ScienCell).

### Small interfering RNAs (siRNA)

U87MG cells growing in 6-well plates were incubated with human siRNAs (100 nmol/L) for H3R/Akt2/ERK1. Mock-transfection was performed using a negative control siRNA (Santa Cruz Biotechnology) as control. Cells were harvested at 48 h post transfection, washed and stored for future experiments. The knockdown efficiency was assessed using the western blot assay.

### Cell proliferation assay

Cells were plated in 96-well plates (1.5 × 10^4^ cells/well) for 24 h. At this time, 10 μL CCK-8 reagent (Dojindo) was added to each well and then the plates were incubated at 37°C for 90 min. Absorbance was measured using a Microplate Reader (Bio-Rad).

### Wound healing migration assay

The siH3R U87MG cells and siNC U87MG cells were seeded in 6-well dishes and cultured under starvation conditions overnight until they reached 80%–90% confluence. A sterile one-milliliter pipette tip was used to generate a wound across the cell monolayer, and the debris was washed with PBS. In parallel, U87MG cells were were treated with an H3R agonist/antagonist. After 48 h, cells migrating into the wounded area or protruding from the border of the wound were visualized, photographed and counted under the inverted microscope. A total of nine areas were selected randomly in each well using a 100× magnification and cells in three wells of each group were counted for each experiment. Experiments were carried out in triplicate for a minimum of three times.

### Invasion assay

The upper chamber of a 24-well hanging cell culture insert was pre-coated with 24 μg/μl Matrigel (BD Biosciences). 5 × 10^5^ U87MG cells were resuspended in 200 μL serum-free media and seeded onto the upper chamber of a 24-well hanging cell culture insert (Millipore) fitted with polyethylene terephthalate (8.0 mm pore size). The agonist/antagonists for H3R, PI3K, MEK or the G_i/o_ protein (all from Santa Cruz Biotechnology) were then added to the upper chamber as follows (100 μmol/L LY294002 for PI3K; 10 μmol/L U-0126 for MEK; 100 ng/mL PTX for G_i/o_ protein) and 900 μl DMEM media with 20% FBS was added to lower chamber of each well. After 48 h, cells were fixed using 4% paraformaldehyde and dyed with crystal violet. Cells on the top surface of the insert were removed with a cotton swab. Cells adhering to the lower surface were fixed with methanol, stained with Giemsa solution and counted under a microscope in five predetermined fields (200×).

### Xenograft tumor establishment

All animal procedures were performed following the National Animal Experimentation guidelines and were approved by the ethics committee of the Fourth Military Medical University. All mice were bred in aseptic conditions at a constant humidity and temperature with standard 12 h light-dark cycles and free access to drinking water and standard chow. U87MG cells (1 × 10^7^ in 100 mL of saline) were suspended in a volume of 0.2 ml serum-free medium and injected subcutaneously into the hind flanks of the animals. At the end of the treatment, the nude rats were sacrificed and tumor volumes were calculated (0.5 × length (mm) × width^2^ (mm^2^). Tumors were harvested, fixed in formalin and embedded in paraffin.

### Immunohistochemistry assays

The pathology specimens were quickly removed, post fixed overnight at 4°C, dehydrated in graded sucrose and processed for embedding in paraffin wax with the anatomical orientation preserved. Sections 3–4 μm in thickness were mounted on silane-coated slides and air-dried. Before immunohistochemistry staining, sections were placed in a bathing solution of 3% H_2_O_2_ and 60% methanol PBS (pH 7.4) for 30 min and then treated with 0.01 mol/L sodium citrate buffer at 95°C in a microwave oven for 13 min (antigen retrieval). Specimens were blocked using a 5% normal goat serum and 5% bovine serum albumin in PBS. Before each step, sections were rinsed three times in PBS buffer. Incubation with H3R primary antibodies (1:100, Abcam) was performed in a PBS-based solution of 1% bovine serum albumin for 12 h at 4°C in the recommended dilutions. After rinsing with PBS, sections were incubated with the corresponding secondary biotinylated antibodies for 1 h at room temperature. A streptavidin/horseradish peroxidase complex was then applied as a detection system for 1 h (1:100; Abcam). Finally, staining was developed using 3, 3′diaminobenzidine tetra-hydrochloride in 0.05 mol/L Tris-HCl buffer and 0.1% H_2_O_2_. Negative control sections were incubated without the primary antibody.

### Image analysis

Image analysis was performed using a DMR-X microscope coupled to a DC500 digital camera (Leica) and the image analysis system Quantimet Q550 (Leica). Nine randomly selected discontinuous fields (20×) per samples were evaluated. The positive area, total area and the integrated optical density of H3R were stained and quantified using the Image-Pro Plus software (Media Cybernetics). The PEA was measured as positive area/total area × 100%. The MD of H3R expression in a selected discontinuous fields was measured as the integrated optical density/total area × 1000. The average of PEA and MD form the above nine fields were calculated as the behalf of a sample for the further data analysis.

### Real-time PCR analysis

Tissue samples and cells were put into liquid nitrogen for 10 min and then into an ultra freezer at 80°C. Total RNA was extracted from glioma cell lines and sample tissues using the Trizol reagent according to the manufacturer's instructions (Takara). RNA was transcribed to cDNA using the PrimeScript RT reagent Kit (Takara). For H3R expression analysis, the products were separated by electrophoresis on 1.5% (v/v) agarose gels, stained with ethidium bromide, and visualized under UV light. The qRT-PCR for other investigated genes was performed using the CFX96TM real-time system (Bio-Rad), and the relative gene expression was normalized to the internal control β-actin. The primers used were as follows: H3R: Forward: 5′-TCATCGTGAGCATCTTTGGG-3′, Reverse: 5′-ACAGAGGGTAGAGG ACAGGGTT-3′; Snail: Forward: 5′-TTTACCTTCCAGCAGCCCTA-3′, Reverse: 5′-GACAGAGTCCCAGATGAGCA-3′; Slug: Forward: 5′-ACAGCGAACTGGAC ACACAT-3′, Reverse: 5′-GGAATGGAGCAGCGGTAGT-3′; Twist: Forward: 5′-CACCATCCTCACACCTCTGC-3′, Reverse: 5′-GCTGATTGGCACGACCTCT-3′.

### Western blot assay

Proteins were extracted from tissue samples or cells. Total protein concentration was determined using a bicinchoninic acid (BCA) kit for protein determination (Takara). The samples were resolved in 10% SDS-PAGE gels and transferred to polyvinylidene fluoride (PVDF) membranes. Immunoblots were probed with primary antibodies overnight at 4°C. The following antibodies used were from Abcam: H3R (1:1000), E-cadherin (1:10000), N-cadherin (1:5000), ZO-1 (1:50), vimentin (1:1000), phospho-PI3K (Tyr607)(1:500). The following antibodies were from Cell Signaling: phospho-Akt (Ser473) (1:1000), Akt (1:1000), phospho-ERK1/2 (Thr202/Tyr204)(1:1000), ERK1/2 (1:1000), phospho-MEK1/2 (Ser217/Ser221)(1:1000), MEK1/2 (1:1000), PI3K (1:1000) and β-actin (1:1000). All primary antibodies were used according to the instructions provided by the supplier. After that, the membrane was incubated with the corresponding HRP-linked anti-rabbit IgG antibody (1:2000, Cell signaling) at room temperature for 1 h. The blots were visualized using the ECL-Plus reagent (Millipore).

### Statistical analysis

SPSS 16.0 (SPSS Inc) and Graph Pad Prism 5.0 software (Graph Pad Software) were used to analyze the data and all values were expressed as mean ± SD. Statistical analysis was performed using one-way ANOVA (Analysis of Variance) followed by Bonferroni's multiple comparison tests where appropriate. The Mann-Whitney *U* test was used for data with heterogeneity of variance. A value of *P* < 0.05 was considered significant.
